# Alterations in articular cartilage T2 star relaxation time following mechanical disorders: in vivo canine supraspinatus tendon resection models

**DOI:** 10.1186/s12891-020-03447-3

**Published:** 2020-07-02

**Authors:** Dokwan Lee, Ki-Taek Hong, Tae Seong Lim, Eugene Lee, Ye Hyun Lee, Ji Soon Park, Woo Kim, Joo Han Oh, Jung-Ah Choi, Yongnam Song

**Affiliations:** 1 Department of Mechanical Engineering, Korea University Engineering Campus, Innovation Hall, Room 306, Anam-dong, Seongbuk-gu, Seoul, 02841 South Korea; 2 Department of Radiology, Gachon University Gil Medical Center, Incheon, South Korea; 3 Department of Radiology, Seoul National University Bundang Hospital, Seongnam, South Korea; 4 Department of Orthopedic Surgery, National Police Hospital, Seoul, South Korea; 5 Department of Orthopedic Surgery, Sheikh Khalifa Specialty Hospital, Ras Al Khaimah, United Arab Emirates; 6 Seoul Kiwoonchan Orthopedics Clinic, Seoul, South Korea; 7 Department of Orthopedic Surgery, Seoul National University Bundang Hospital, Seongnam, South Korea; 8 Department of Radiology, Hallym University Dongtan Sacred Heart Hospital, Hwaseong, South Korea

**Keywords:** Articular cartilage, Magnetic resonance imaging, Osteoarthritis, Biomechanics, Animal model

## Abstract

**Background:**

The role of altered joint mechanics on cartilage degeneration in in vivo models has not been studied successfully due to a lack of pre-injury information. We aimed 1) to develop an accurate in vivo canine model to measure the changes in joint loading and T2 star (T2*) relaxation time before and after unilateral supraspinatus tendon resections, and 2) to find the relationship between regional variations in articular cartilage loading patterns and T2* relaxation time distributions.

**Methods:**

Rigid markers were implanted in the scapula and humerus of tested dogs. The movement of the shoulder bones were measured by a motion tracking system during normal gaits. In vivo cartilage contact strain was measured by aligning 3D shoulder models with the motion tracking data. Articular cartilage T2* relaxation times were measured by quantitative MRI scans. Articular cartilage contact strain and T2* relaxation time were compared in the shoulders before and 3 months after the supraspinatus tendon resections.

**Results:**

Excellent accuracy and reproducibility were found in our in vivo contact strain measurements with less than 1% errors. Changes in articular cartilage contact strain exhibited similar patterns with the changes in the T2* relaxation time after resection surgeries. Regional changes in the articular cartilage T2* relaxation time exhibited positive correlations with regional contact strain variations 3 months after the supraspinatus resection surgeries.

**Conclusion:**

This is the first study to measure in vivo articular cartilage contact strains with high accuracy and reproducibility. Positive correlations between contact strain and T2* relaxation time suggest that the articular cartilage extracellular matrix may responds to mechanical changes in local areas.

## Background

Articular cartilage is known to be modulated by mechanical loading. Changes in loading conditions have been reported to alter the structural and compositional characteristics of articular cartilage, and may initiate osteoarthritis (OA) development [1–3]. Previous studies have shown that the biochemical composition of articular cartilage varies across different regions and is related to regional loading history [4–6]. Weight-bearing regions of articular cartilage exhibit greater proteoglycan content compared to non-weight-bearing articular cartilage, whereas collagen content has been found to be greater in non-weight-bearing areas [5–8]. Because proteoglycan and collagen networks are crucial for maintaining the mechanical strength of articular cartilage, the conditions of proteoglycan content and collagen networks can provide valuable information for estimating the degree of articular cartilage degeneration [9–11].

Traditional measurements of articular cartilage compositional properties are based on destructive biochemical and histological procedures that are not applicable for clinical diagnostic purposes. Recently, T1 and T2 relaxation properties of quantitative magnetic resonance imaging (qMRI) have been proposed for estimating articular cartilage biochemical compositions. Because qMRI utilizes the magnetic relaxation characteristics of water components and macromolecules, magnetic relaxation parameters (T1, T1ρ, and T2) are believed to reflect the tissue hydration, collagen, and proteoglycan compositions of articular cartilage [12–16]. Among these relaxation values, T2 relaxation time has been widely used to examine the degeneration of articular cartilage tissues [12, 17–19]. Prolonged T2 relaxation time is believed to be a sign of degenerative articular cartilage, which indicates damage to collagen networks and increased water content in articular cartilage extracellular matrices [18–20].

Many studies have investigated the relationship between T2 relaxation time and loading patterns [21–23]. Significant unloading of knee joints resulted in increased T2 relaxation time, but T2 relaxation time returned to the normal level when the knee resumed natural weight-bearing activities [21]. Mild exercises with low-level shear and compressive loadings have been reported to effectively reduce the T2 relaxation time of articular cartilage in the early stages of OA [22, 23]. These studies indicate that mild mechanical loadings are important for maintaining healthy articular cartilage whereas a large magnitude of mechanical loading or unloading is detrimental to articular cartilage. Articular cartilage T2 relaxation time measurements have also been used to estimate articular cartilage conditions following joint injuries in various clinical studies. The cartilage T2 relaxation times in knees with anterior cruciate ligament (ACL) tears or reconstructed knees were found to be greater than the times in intact knees [24]. Because ACL injuries significantly alter joint biomechanics [25, 26], changes in T2 relaxation time may be the consequence of mechanically induced articular cartilage degradation. Recently, T2 star (T2*) relaxation time has been widely examined as an alternative method of T2 relaxation time measurements because T2* relaxation time have been reported to positively correlate with T2 relaxation time values on the estimation of articular cartilage degeneration [27, 28]. Additionally, the short acquisition time, high signal-to-noise ratio (SNR), and high out-of-plane resolutions of T2* imaging procedures should be significant advantages in clinical imaging situations [16, 27].

In previous clinical studies, mechanically induced articular cartilage degeneration and T2 relaxation time has been investigated by comparing stress/strain patterns and T2 relaxation time distributions between the joints with injuries and the contralateral intact joints, while the cartilage conditions prior to injuries were not available in most cases [29, 30]. However, it is important to study the changes in joint mechanics and T2 relaxation time before and after injuries in a joint to completely understand the role of mechanical alterations on articular cartilage degenerations because articular cartilage biochemical and biomechanical properties might be different between contralateral joints even in the same individual [31]. In this study, we aimed to develop an accurate in vivo canine model to measure the changes in joint loading and T2* relaxation time before and after supraspinatus tendon tears. We measured regional variations in articular cartilage loading patterns and T2* relaxation time distributions after supraspinatus resections. We hypothesized that supraspinatus tendon tears would alter the loading patterns and T2* relaxation time distributions in the shoulder articular cartilages. We also hypothesized that spatial variations in joint loading and T2* relaxation time would be correlated. 

## Methods

### Ethical statement and study design

This study was approved by the Animal Care and Use Committee (approval number: BA1507–180/047–01) and carried out in accordance with the Guide for the Care and Use of Laboratory Animals of the Seoul National University College of Medicine (Republic of Korea). Total six shoulders from three adolescent mongrel dogs (approximately one year old and weighed 20 kg) were examined. Dogs were obtained from Kukje Laboratory Animal Center (Republic of Korea). After the completion of the study, the animals were euthanized with intraventricular administration of potassium chloride (2 mmol/kg) under deep anesthesia by following AVMA (American Veterinary Medical Association) guidelines.

### Experimental procedure and experimental animals

#### Gait analysis

In this study, we measured articular cartilage deformation patterns by using kinematics-based 3 dimensional (3D) shoulder models. We decided to use a marker-based motion tracking system (eight infrared cameras with a sampling frequency of 120 frames/sec, Eagle Digital real-time system, MotionAnalysis Co., USA). The large tracking areas of motion tracking systems are suitable for normal walking activities in in vivo canine models.

**Fig. 1:**
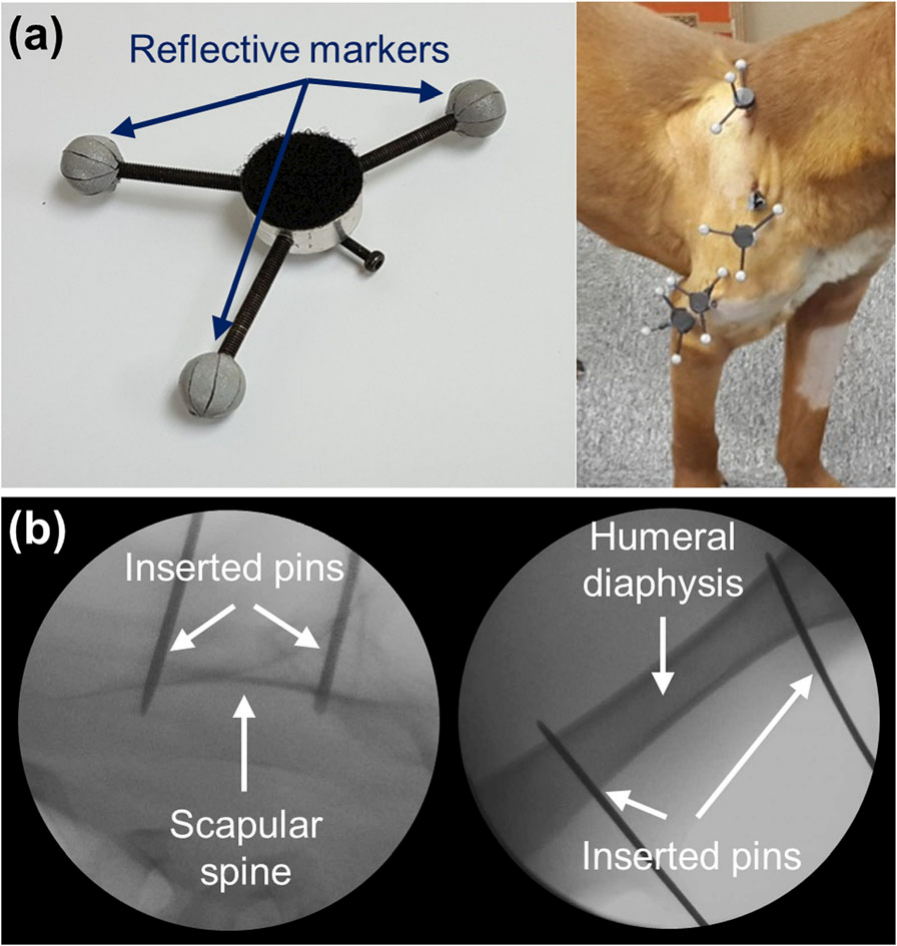
A custom marker and its installation to shoulder bones. **a** A custom marker was designed with three conventional reflective balls. **b** Two custom markers were inserted along the scapular spine and other two markers were inserted into the distal and proximal ends of the humerus along the lateral side of the diaphysis

We decided to implant custom markers into shoulder bones rigidly to minimize skin artifacts in the motion tracking data. A custom marker was designed with three conventional reflective balls (diameter of 8 mm) (Fig. 1a). Two custom markers were installed in the humerus and scapula using orthopedic stainless surgical threaded pins. Scapular pins were inserted in two locations along the scapular spine and humeral pins were inserted into the distal and proximal ends of the humerus along the lateral side of the diaphysis (Fig. 1b). After four to 5 days of recovery following the marker insertion surgeries, we recorded the movements of the shoulder bones by tracking the ball markers as the dogs walked freely within our motion laboratory. Only one shoulder was tested in a single motion analysis examination to minimize the loosening of surgical pins. We found that the pins in shoulder bones were easily loosen when the tested dog laid the pin-inserted shoulder on the floor. Thus, the contralateral shoulder remained intact to allow the tested dog to use the intact shoulder in resting positions. After 2 weeks of recovery time, gait analysis of the contralateral shoulder was followed thorough the identical marker installation and motion tracking procedures.

#### 3D shoulder models

**Fig. 2:**
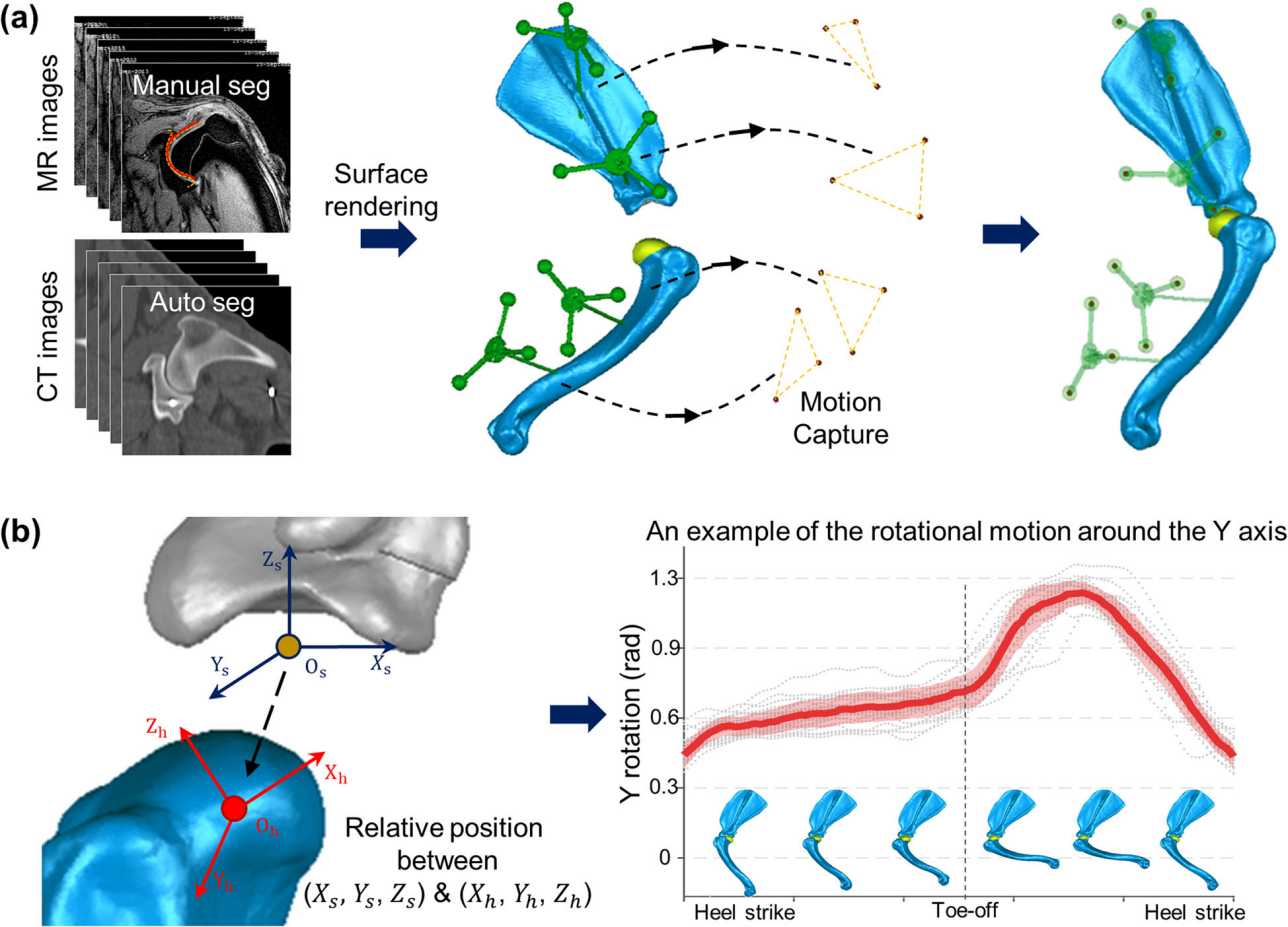
Registration of 3D shoulder models to motion analysis data. **a** Image-based 3D shoulder models with scapular and humeral bones, articular cartilage, and custom markers were aligned with the marker positions from the motion tracking data. **b** The relative positions between the centers of the humeral head and glenoid cavity were calculated with six degrees of freedom motions (an example of the rotational motion between the Y axis of the humeral head and glenoid cavity)

The 3D shoulder models were created from computed tomography (CT) (matrix: 512 × 512, field of view (FOV): 400 mm, slice thickness: 2.2 mm, slice spacing: 1 mm, Brilliance CT 64-channel, Philips, Netherlands) and magnetic resonance (MR) (T2 weighted fast field echo (FFE) sequence, repetition time (TR): 575 ms, echo time (TE): 11.51 ms, flip angle: 20°, matrix: 512 × 512, FOV: 130 mm, slice thickness: 2 mm, slice spacing: 2.2 mm, Achieva 3.0 T TX, Philips, Netherlands) images of the shoulder bones of tested dogs. We tried to maintain the location and orientation of shoulder joints in each imaging step to minimize potential artifacts in MR images which might be generated from the magic angle effect [32]. The shoulder bones and custom markers were automatically reconstructed from the CT images using the OSIRIX (Pixmeo, Switzerland) and GeoMagic (Research Triangle Park, NC, USA) software, while the articular cartilage layers were manually segmented from the MR images using a custom MATLAB code. The 3D bone and cartilage models were then combined by aligning the subchondral bone profiles of each model (best-fit alignment in GeoMagic software). The final shoulder models included scapular and humeral bones, articular cartilage, and custom markers (Fig. 2a).

#### Supraspinatus tendon resection model

After completing articular cartilage contact strain and T2* relaxation time maps for intact shoulders, we completely resected the supraspinatus tendon in one of the shoulders while the contralateral shoulder remained intact. The resection area was covered with penrose drain tubes to prevent a recovery of the resected muscle. The dogs were allowed to perform normal activities in our animal research laboratory for 3 months following the resection surgeries (cage type: SUS304 stainless steel frame cage with fiber reinforced plastic (FRP) bottom plate (3.4 m × 1.3 m × 2.4 m), one dog per each cage with automatic watering system, temperature: 20 ± 2 °C, humidity: 50 ± 10%, light cycle (12 h): 7 am to 7 pm). Measurements of the articular cartilage contact strain and T2* relaxation time patterns were then repeated for both shoulders in the supraspinatus-resected dogs. All surgical processes were conducted under general anesthesia with an intramuscular injection of 1) satropine sulfate (0.1 ml/kg of body weight, DAI HAN PHARM. Co., Ltd., Seoul, Korea) and 2) the mixture (0.2 ml/kg of body weight) of xylazine (Rompun, Bayer Korea, Seoul, Korea) and tiletamine-zolazepam (Zoletil 50, Virbac, Carros, France).

### Experimental outcomes

#### Gait pattern and articular cartilage strain measurement

The completed 3D shoulder models were aligned with the marker positions from the motion tracking data to determine the locations of shoulder bones in each gait frame (Fig. 2a). The relative positions between the centers of the humeral head and glenoid cavity were calculated with six degrees of freedom (DOFs) (three translational and three rotational motions in a Cartesian coordinate system) (Fig. 2b). An example of the rotational motion between the Y axis of the humeral head and glenoid cavity is shown in Fig. 2b. We removed any abnormal gait cycles that were outside of the mean ± one standard deviation (red shaded area in Fig. 2b) from all six DOF components. The remaining gait cycles were then averaged to generate a representative gait pattern for each dog (solid red line in Fig. 2b).

**Fig. 3:**
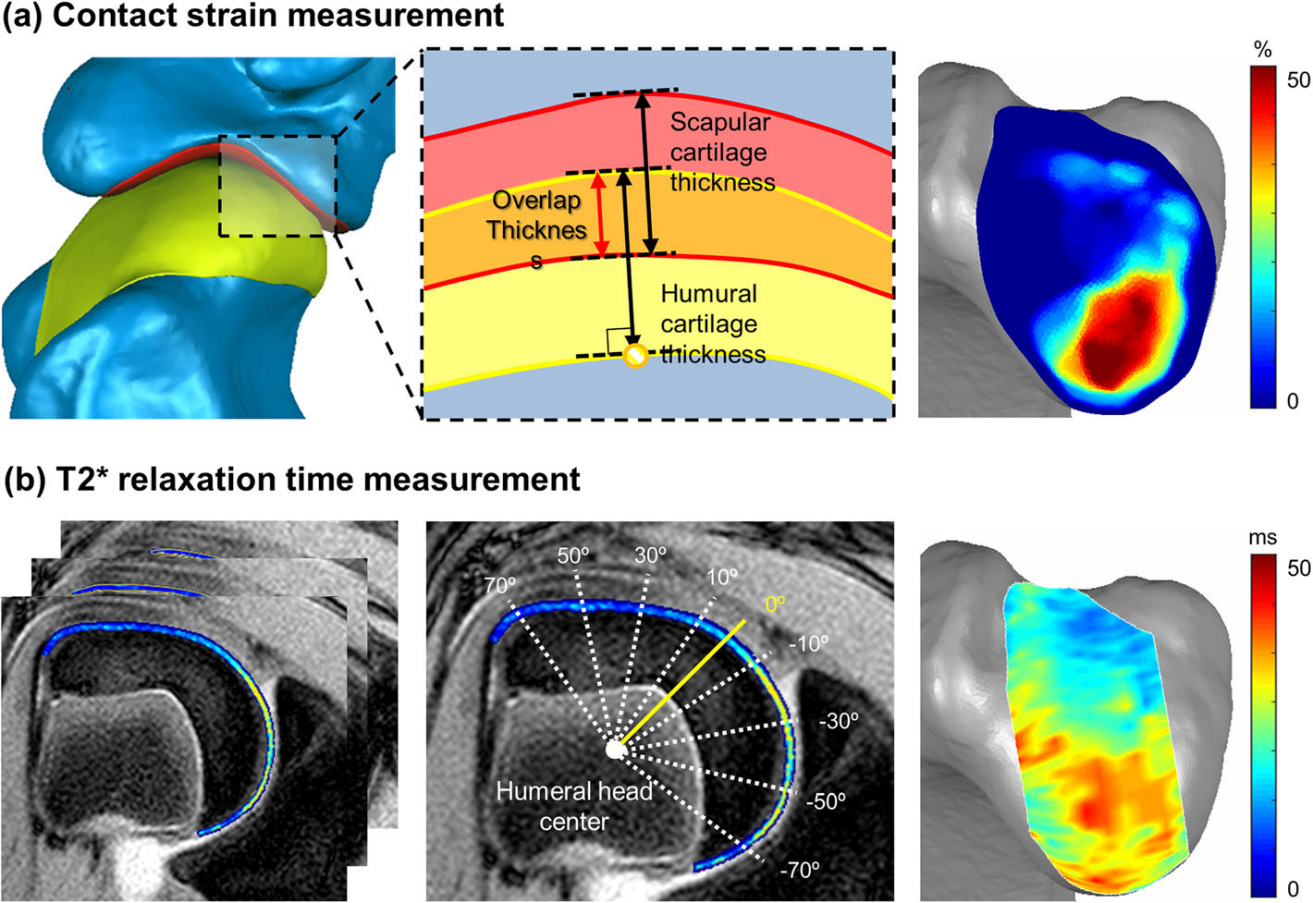
3D maps of articular cartilage contact strain and T2* relaxation time. **a** Articular cartilage contact strain was defined as the ratio between the undeformed articular cartilage thickness and the thickness of the overlapping areas between the scapular and humeral articular cartilage. **b** The T2* relaxation time of each pixel in the MR images was calculated and each articular cartilage surface was divided into multiple anterior-to-posterior 20° regions

Articular cartilage thickness was determined by calculating the perpendicular distance from the subchondral bone interface to the articular cartilage surface for both the scapular and humeral cartilage. Articular cartilage contact strain was defined as the ratio between the undeformed articular cartilage thickness and the thickness of the overlapping areas between the scapular and humeral articular cartilage in each gait fame (Fig. 3a) [33, 34]. The articular cartilage contact areas were then defined as the cartilage areas in which the overlapping articular cartilage thickness was greater than 0.25 mm (in-plane resolution of the MR images). We created a 3D articular cartilage contact strain map for each gait frame from an entire representative gait cycle and combined all of these strain maps to generate a cumulative contact strain distribution. In this cumulative strain map, a cumulative contact area was the combination of individual contact areas from each gait frame.

#### Articular cartilage T2 star (T2*) relaxation time measurement

A qMRI scan of each tested dog was performed on the same day as the morphological CT and MR imaging. A T2 weighted multi-echo fast-field sequence (TR: 700 ms, TE: 3.83/9.37/14.91/20.46/26.01 ms, flip Angle: 25°, matrix: 768 × 768, FOV: 150 mm, slice thickness: 2 mm, slice spacing: 2.2 mm) was used in a 3 T clinical MRI scanner (Achieva 3.0 T TX, Philips, Netherlands). The T2* relaxation time of each pixel in the MR images was calculated using mono-exponential least squares fitting [35]. T2* relaxation time values over 50 ms were removed to minimize the partial effects of synovial fluid around articular cartilage boundaries because T2 relaxation time of synovial fluid is known to be approximately 100 ms [36], and the T2* relaxation time is known to be around 50% of the T2 relaxation time values [27]. We divided each articular cartilage surface into multiple 20° regions from the posterior to the anterior ends (Fig. 3b) because our contact strain maps indicated anterior-to-posterior directional variations after the resection surgeries, but the changes in the medial-to-lateral direction were minimal.

#### Accuracy and reproducibility of contact strain measurements

Intra- and inter-observer reliability of manually segmented articular cartilage models was tested. Segmentation of articular cartilage surface and subchondral bone interface in a shoulder joint was done by three independent researchers in three difference days. Multiple 3 dimensional articular cartilage models were generated from various segmentation results for a shoulder joint. Articular cartilage thickness values at 70 random locations were compared in different cartilage models. Intra-class correlation coefficients (ICC) with 95% confident interval (CI) and average root-mean-square (RMS) differences in the thickness measurements among different segmentation results were calculated. We also calculated variations in average T2* relaxation time when articular cartilage thickness was increased or decreased within the average RMS difference in the thickness measurements.

**Fig. 4:**
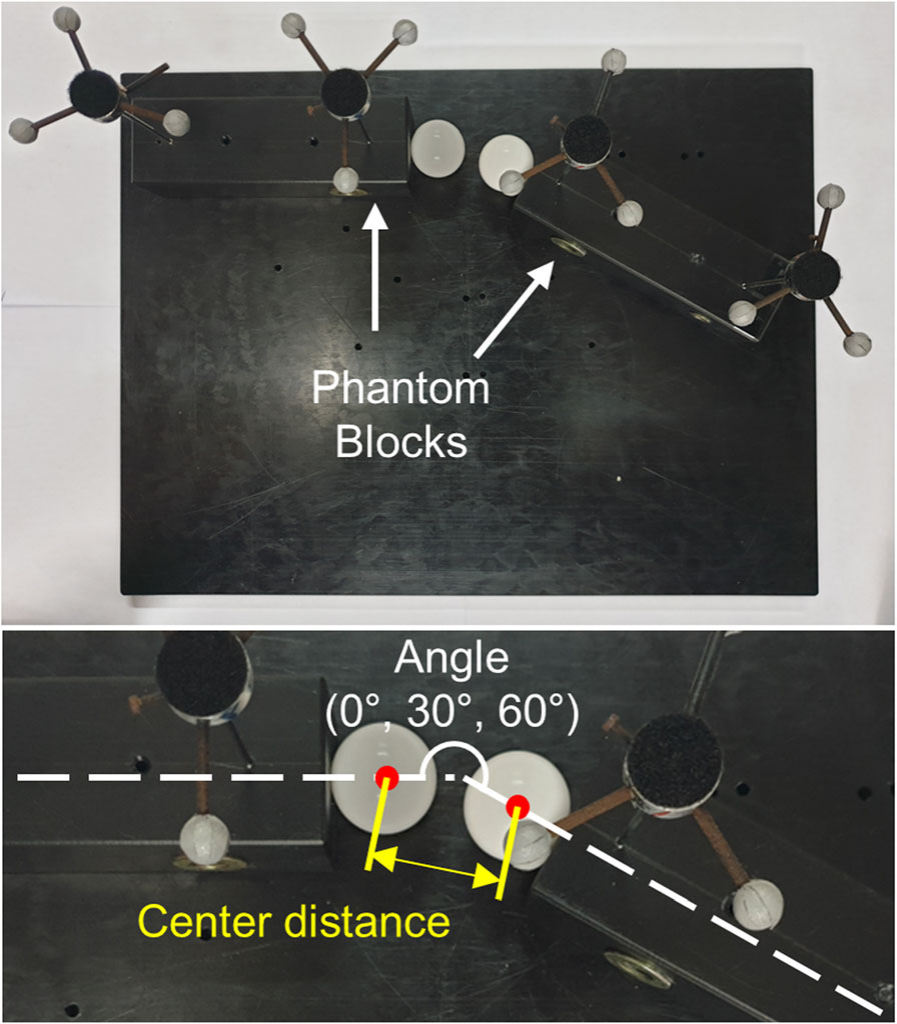
A phantom model for the accuracy and reproducibility evaluation. The phantom consisted of two rectangular blocks with a plastic ball attached at one end of each block (three different block angles of 0°, 30°, 60°)

Because articular cartilage contact strains were directly determined based on the location of the scapular and humeral bones, we decided to measure the accuracy and reproducibility of our motion-tracking-based 3D shoulder models using a custom plastic phantom model. The phantom consisted of two rectangular blocks with a plastic ball attached at one end of each block. The phantom blocks were positioned in three different configurations (relative angles of 0°, 30°, 60°). Two custom markers were installed in each phantom block to emulate the in vivo experiments (Fig. 4). We moved the phantom model in the motion laboratory in random directions for 10 min while the motion tracking system continuously recorded the positions of the markers. We created a 3D model of the phantom blocks and measured 1) the center distance between the two phantom balls and 2) the angle between the two phantom blocks for 2000 randomly selected frames. The bias (average differences in the motion-tracking-based measurements from the physical measurements of the phantom model) and precision (variations in the motion-tracking-based measurements) of the center distance and angle measurements were then calculated to find systemic errors in motion tracking system. Finally, corresponding bias and precision of articular cartilage contact strain and contact area ratio (ratio between contact area and total cartilage surface area) in in vivo shoulder models were estimated by changing the position of scapular and humeral bones within the range of bias and precision values measured in the phantom experiments at each configuration (0°, 30°, 60° angles between scapula and humerus).

### Statistical methods

Intra- and inter-observer ICC with 95% confidence interval (CI) of segmentations were analyzed by using SPSS software (SPSS statistics 25, SPSS Inc., IL, USA). Pearson correlation coefficients between articular cartilage contact strain and T2* relaxation time were calculated by using MATLAB software (R2016a, MathWorks, MA, USA). A *p*-value less than 0.05 was considered to be a statistically significant difference.

## Results

### Accuracy and reproducibility of contact strain measurements

Excellent intra- and inter-observer reliability of cartilage thickness measurements in different segmentations were found with ICCs over 0.850. Average RMS differences in the thickness measurements between cartilage models from 3 different days and observers were 0.097 mm and 0.125 mm respectively (Table 1). Variations in the average T2* relaxation time were 0.496 ± 0.202 ms when the articular cartilage thickness varied within 0.125 mm which was the maximum value among intra- and inter-observer RMS differences in cartilage thickness measurements.

**Table 1:** Intra- and inter-observer reliability of cartilage thickness measurements

	ICC (95% CI)	RMS difference
Intra-observer reliability	0.917 (95% CI: 0.901, 0.931)	0.097 ± 0.074 mm
Inter-observer reliability	0.874 (95% CI: 0.849, 0.895)	0.125 ± 0.067 mm

Intra-class correlation coefficients (ICC) with 95% confident interval (CI) and average root-mean-square (RMS) differences in the thickness measurements between different segmentation results were calculated.

The bias and precision of motion-tracking-guided measurements in the phantom were less than 0.050 mm for the center distance and 0.692 degree for the angle measurements (Table 2). Estimated errors (bias) in the articular cartilage contact strain and contact area ratio were found to be less than 0.349 and 0.570% respectively. Variations in the measurements (precision) were less than 0.696% for average contact strains and 1.020% for contact area ratios (Table 2).

**Table 2:** Bias and precision of motion-tracking-guided measurements

	Phantom model				In vivo shoulder model			
	Center distance (mm)		Block angle (°)		Average contact strain (%)		Contact area ratio (%)	
	*Bias*	*Precision*	*Bias*	*Precision*	*Bias*	*Precision*	*Bias*	*Precision*
Position 1 (Angle: 0°)	0.006	+ 0.089 – 0.089	0.049	+ 0.133 – 0.133	0.066	– 0.484 + 0.575	0.048	– 1.020 + 0.947
Position 2 (Angle: 30°)	– 0.023	+ 0.056 – 0.056	– 0.692	+ 0.126 – 0.126	– 0.138	– 0.425 + 0.465	– 0.308	– 0.850 + 0.758
Position 3 (Angle: 60°)	– 0.050	+ 0.084 – 0.084	0.027	+ 0.136 – 0.136	– 0.349	– 0.604 + 0.696	– 0.570	– 0.929 + 0.997

The bias and precision of the center distance and angle measurements in the phantom model are listed, and corresponding bias and precision of articular cartilage contact strain and contact area ratio in in vivo shoulder models are also estimated.

### Variations in articular cartilage contact strain and T2* relaxation time after supraspinatus resection

**Fig. 5:**
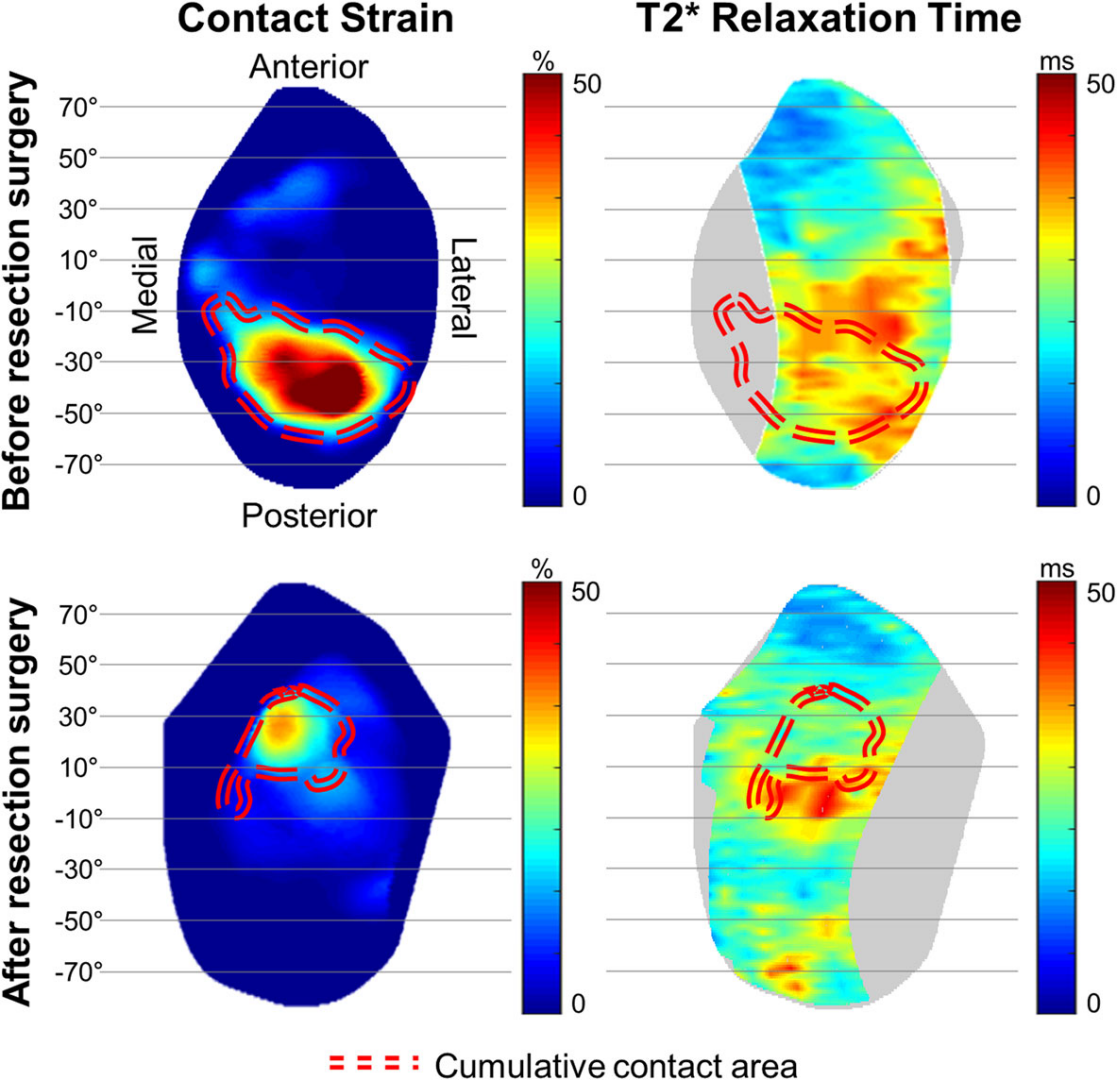
Changes in cumulative contact strain and T2* relaxation time distributions after a supraspinatus resection surgery. An example of the articular cartilage cumulative contact strain and T2* relaxation time patterns before and after the supraspinatus resection surgery for one of the tested shoulders are presented

**Fig. 6:**
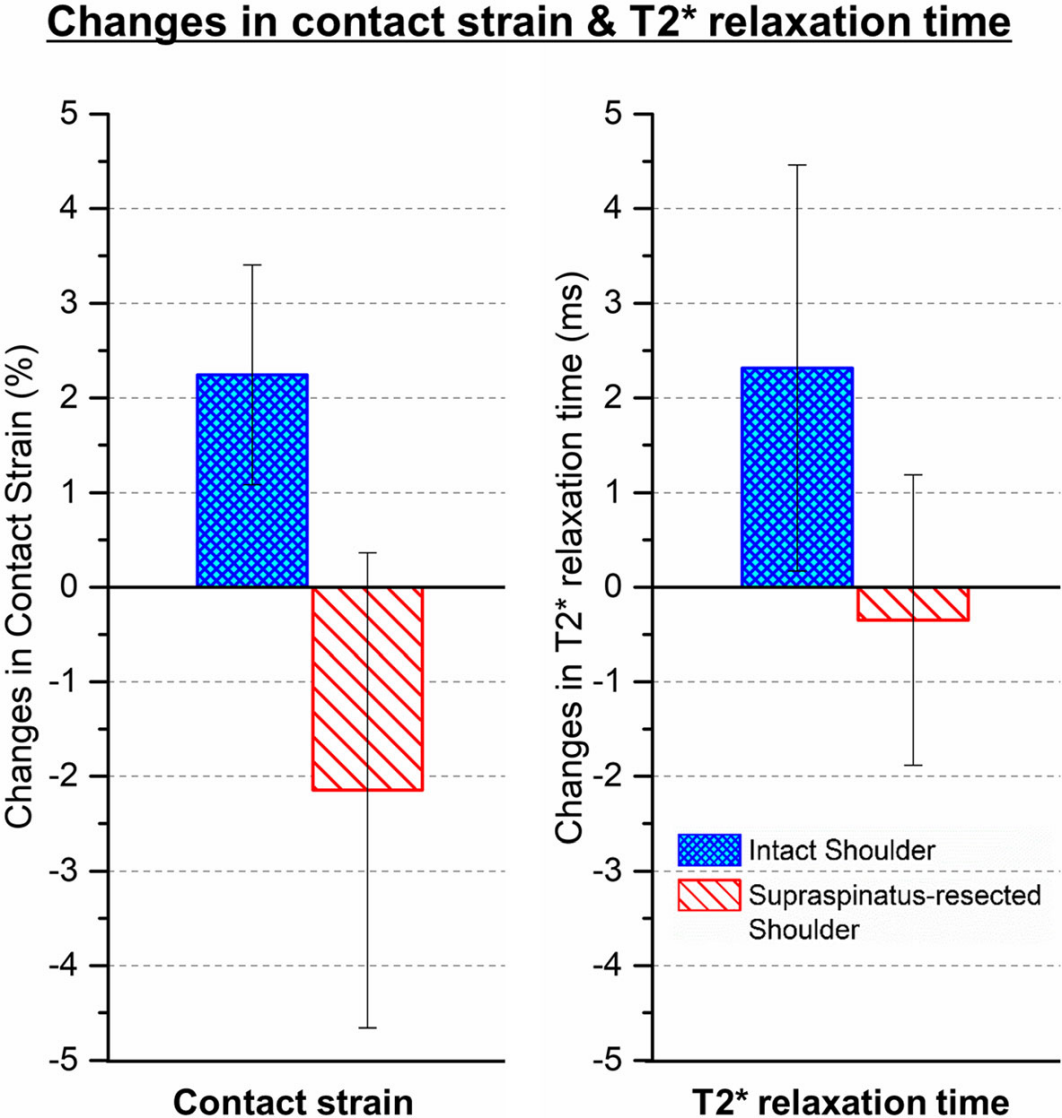
Changes in average cumulative contact strain and T2* relaxation time after supraspinatus resections. Changes in the average articular cartilage cumulative contact strain and in the average T2* relaxation time are shown after the supraspinatus resection surgeries

In the 3D shoulder models of all tested dogs, average and maximum thickness values of articular cartilage were measured at 0.819 ± 0.086 mm and 1.227 ± 0.166 mm respectively. Unilateral supraspinatus resection surgeries were found to alter articular cartilage contact strain patterns in both shoulders 3 months after the supraspinatus resection surgeries. An example of the articular cartilage cumulative contact strain patterns for one of the tested shoulders is presented in Fig. 5. The average articular cartilage cumulative contact strain was reduced in the supraspinatus-resected shoulders by approximately 2.145%. However, the average cumulative contact strain in the contralateral shoulders increased by approximately 2.243% (Fig. 6).

Average T2* relaxation time variations after the supraspinatus resection surgery exhibited similar patterns with the patterns in the average cumulative contact strain. The average articular cartilage T2* relaxation time in the supraspinatus-resected shoulders decreased, whereas the T2* relaxation time in the intact shoulders increased over 2.316 ms after the resection surgeries (Fig. 6).

### Regional changes in contact strain and T2* relaxation time

**Fig. 7:**
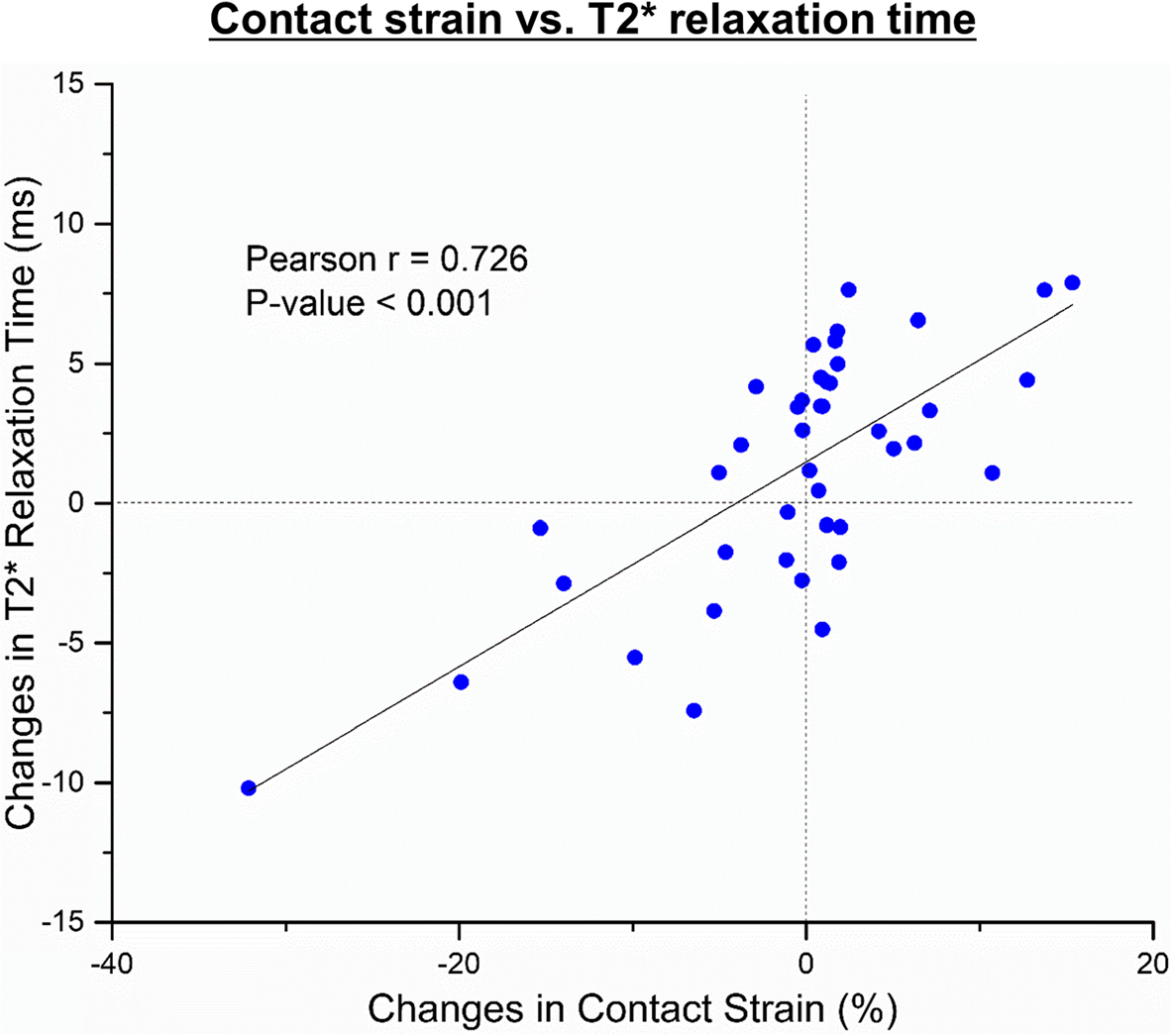
Correlation between regional changes in contact strain and T2* relaxation time following the supraspinatus resections. The regional variations in articular cartilage contact strain and T2* relaxation time exhibited a linear correlation in both the intact and supraspinatus-resected shoulders

Because cartilage contact strain and T2* relaxation time were found to concentrate in local contact areas (Fig. 5), we decided to calculate regional correlations between the changes in articular cartilage contact strain and T2* relaxation time following the supraspinatus resections in each of the anterior-to-posterior 20° regions for all tested shoulders. The regional variations in articular cartilage contact strain and T2* relaxation time exhibited a positive linear correlation with a Pearson correlation coefficient (r) of 0.726 (*p* < 0.001, Fig. 7).

## Discussion

Injury-related articular cartilage degeneration is believed to be the consequence of mechanical alterations. However, the role of altered joint mechanics in articular cartilage degeneration in in vivo models has not been studied successfully due to the lack of information regarding pre-injury articular cartilage conditions. This is the first in vivo study to compare articular cartilage contact strains and T2 star (T2*) relaxation times before and after a joint injury. We successfully developed a technique for measuring in vivo articular cartilage contact strains using a motion tracking system and image-based 3D shoulder models with high accuracy and reproducibility. Unilateral supraspinatus tendon resection altered articular cartilage contact patterns in both shoulders. Contact strains in supraspinatus-resected shoulders were found to decrease, whereas the contact strains in the contralateral intact shoulders increased. Interestingly, these changes in articular cartilage contact strain exhibited similar patterns with the changes in the articular cartilage T2* relaxation time following the supraspinatus resection surgeries throughout all tested shoulders (Fig. 6). It was further supported by positive correlations between regional variations in articular cartilage contact strain and T2* relaxation time (Fig. 7). These results are consistent with previous clinical studies which reported increased T2* relaxation time on the regions of increased articular cartilage contact loads [37–39]. Although those clinical measurements must be the result of long-term response to altered joint loading, our relatively short three-month study suggests that the changes in the articular cartilage extracellular matrix may be initiated at the early stage of load variations.

Because articular cartilage contact strains were calculated directly from position measurements of the shoulder bones, we attempted to estimate the accuracy and reproducibility of the position measurements of the proposed motion-tracking-derived 3D models using a plastic phantom. The errors of the position measurements were less than 50 μm in terms of translation and less than 0.7° in terms of angular displacement for all three configurations. The errors in the phantom experiments were expected to generate approximately 0.4% errors in the average contact strain and 0.6% errors in the contact area ratio measurements in in vivo shoulder models. These errors were a combination of systemic errors in the motion tracking device, reconstruction errors in the image-based 3D models, and alignment errors between the motion tracking information and 3D models. The results demonstrate that our technique is as accurate as biplane X-ray radiostereometric analysis, which is typically used for minimized and limited joint motions (accuracy of approximately 10–250 μm in terms of translation and 0.03–0.6° in terms of angular displacement) [40, 41]. The accuracy of our method was found to be better than the accuracy of the popular 2D/3D registration method of biplane fluoroscopy for real-time joint motion analysis (accuracy of approximately 200–700 μm in terms of translation and 0.1–0.9° in terms of angular displacement) [42–44]. Furthermore, our motion-tracking-based method has a wide measurement area, whereas X-ray-based methods have limited measurement space between X-ray sources and detectors. In our in vivo animal study, a large measurement space was crucial to allow natural joint motions without any pre-training.

Unilateral supraspinatus tendon resections altered the articular cartilage contact strain patterns in both shoulders. Decreases in the mean contact strain were found in the supraspinatus-resected shoulders. However, the magnitude of the mean contact strain in the contralateral intact joints markedly increased. This may be the result of compensatory weight shifting behaviors by the dogs to avoid discomfort without losing joint stability following the supraspinatus resection surgeries [45]. T2* relaxation time in the joints with increased contact strain was also increased by approximately 2.5 ms (Fig. 6). Previously, T2* relaxation time in osteoarthritic articular cartilage were reported to be approximately 2 to 5 ms greater than the values in healthy articular cartilage [28, 35, 37]. Although our 2.5 ms increases in T2* relaxation time were smaller than the increases in osteoarthritic cartilages, the increased T2* relaxation time in the region of increased contact strain may indicate the early onset of articular cartilage degenerative processes, which have been identified in abnormally overloaded cartilage areas in various studies [46, 47]. Because articular cartilage T2* relaxation time is related to collagen network integrity and water content in cartilage matrices [12, 16, 48], prominent positive correlations between regional variations in articular cartilage contact strain and T2* relaxation time (Fig. 7) suggests that the articular cartilage extracellular matrices responded to the altered loading patterns, resulting in structural and compositional changes within a relatively short three-month testing period.

This study has a few limitations that must be discussed to contextualize the presented results. First, we only completed six in vivo canine shoulder models from three dogs, despite our original intent of using five dogs for our experiments. One dog died from complications during the anesthesia process for CT scanning. We could not collect motion tracking data for a second dog because the dog did not want to walk in the motion laboratory following the supraspinatus resection surgery. This limited sample size did not allow us to establish various statistical examinations between average contact strain and T2* relaxation time before and after the supraspinatus resections. However, the patterns of increased T2* relaxation time in the shoulders with increased contact strain and decreased T2* relaxation time in the shoulders with decreased strain were consistent for all tested shoulders. The linear correlations between regional contact strain and T2* relaxation time changes in both the intact and supraspinatus-resected shoulders were statistically significant, which supports our hypothesis that alterations in joint mechanics produce important structural and compositional changes in articular cartilage extracellular matrices. Second, the gait patterns recorded by our motion tracking system were not consistent for each gait cycle because joint angles and walking speed varied during normal free walking episodes. Previous studies have shown that a treadmill can produce consistent gait patterns [49]. However, we chose to record the gait cycles of free-walking subjects using a motion tracking system because 1) treadmill walking requires many training sessions for test subjects to become familiar with the system and 2) we wished to avoid any trained gaits using the treadmill, which could have created biased joint mechanics. In this study, we only selected gait cycles from dogs walking in straight lines. We then excluded gait cycles outside of the mean ± one standard deviation range from the selected gaits. The remaining gait cycles were averaged to generate a consistent representative gait pattern. We feel that this final gait pattern successfully described a representative gait motion for each tested dog by including more than 70% of the recorded gait cycles.

## Conclusion

This is the first study to investigate the relationship between articular cartilage contact strain and T2* relaxation time before and after a joint injury in an in vivo animal model. We intentionally created unilateral supraspinatus tendon resections in in vivo canine models to alter joint mechanics, and measured articular cartilage contact strain and T2* relaxation time before and 3 months after the supraspinatus resections. Interestingly, patterns in the articular cartilage contact strain changes were similar to the patterns in the articular cartilage T2* relaxation time variations following supraspinatus resection surgeries. Regional comparisons between articular cartilage contact strain and T2* relaxation time revealed positive correlations in all tested shoulders. Because increased T2* relaxation time is believed to be early signs of articular cartilage degeneration, the degenerative process of articular cartilage may be initiated in areas with increased cartilage strain, potentially leading to the development of osteoarthritis [28, 35].

## Data Availability

The datasets used and/or analyzed during the current study are available from the corresponding author on reasonable request.

## Abbreviations

ACL: Anterior cruciate ligament
CI: Confidential interval;
CT: Computed tomography
DOF: Degrees of freedom
FFE: Fast-field-echo
FOV: Field-of-view
FRP: Fiber reinforced plastic
ICC: Intraclass correlation coefficients
MR: Magnetic resonance
OA: Osteoarthritis
qMRI: Quantitative magnetic resonance imaging
RMS: Root-mean-square
SNR: Signal to noise ratio
TE: Time to echo
3D: Three dimension
TR: Time to repeat
T2*: T2 star
